# The impact of perceived discrimination on subjective health among adolescents

**DOI:** 10.1093/eurpub/ckac130.201

**Published:** 2022-10-25

**Authors:** K Kajikhina, C Koschollek, C Hövener

**Affiliations:** 1 Social Determinants of Health, Rober Koch Institute, Berlin, Germany; 2 Crisis Management, Outbreak Investigations and Training Programmes, Rober Koch Institute, Berlin, Germany

## Abstract

**Background:**

Interpersonal discrimination (disc.) plays an important role for physical and mental health, througoutthe life-course and in particular during adolescence. People can experience disc., e.g. due to their physical appearance or language preferences independently of having a migration background (mb, official statistical category in Germany). Aim of this contribution is to analyse the impact of perceived disc. on subjective health and its relation to mb.

**Methods:**

Using data from from German Health Interview and Examination Survey for Children and Adolescents (KiGGS) wave 2 (2014 - 2017), we analysed by logistic regression the impact of a) overall perceived disc. (≥ one indication of “sometimes” across eight dimensions), b) perceived disc. related to origin, skin colour, accent, language, dialect and c) one- and two-sided mb (one or both parents with own mb) on subjective health among adolescents aged 14 to 17 years.

**Results:**

Among 3,558 adolescents, 21% perceived disc. in at least one of overall eight dimensions, 11% reported disc. related to their origin, skin colour or language. A one-sided mb was measured for 8,4% of participants, a two-sided mb for 20%. Perceiving overall disc. (OR = 3.29) and disc. related to origin, skin colour or language (OR = 1.91) was associated with reporting a (very) bad subjective health. Mb had no impact. Effects remain after controlling for gender, age and socioeconomic position of parents (overall disc. aOR=2.99; disc. related to origin, skin colour or language aOR=1.65).

**Conclusions:**

Perceived disc. is associated with worse subjective health among adolescents, whereas no association with mb is observed. Epidemiological analyses solely focussing on one statistical category such as mb are insufficient as they do not consider those affected by discrimination but are not captured by mb. Differentiated analyses are necessary to elucidate explanatory mechanisms and protective factors.

**Key messages:**

- Discrimination has negative effects on self-rated subjective health.
- Differentiated analyses based on more than “origin” or “migration background” is required to describe public health.

